# 4-Bromo-1-nitro­benzene

**DOI:** 10.1107/S1600536811003394

**Published:** 2011-02-02

**Authors:** Qamar Ali, M. Raza Shah, Seik Weng Ng

**Affiliations:** a H.E.J. Research Institute of Chemistry, International Center for Chemical and Biological Sciences, University of Karachi, Karachi 7527, Pakistan; b Department of Chemistry, University of Malaya, 50603 Kuala Lumpur, Malaysia

## Abstract

The non-H atoms of the title mol­ecule, C_6_H_4_BrNO_2_, are essentially coplanar with an r.m.s. deviation of 0.040 Å. In the crystal, π–π stacking occurs between parallel benzene rings of adjacent mol­ecules with centroid–centroid distances of 3.643 (3) and 3.741 (3) Å. Weak inter­molecular C—H⋯O hydrogen bonding and short Br⋯O contacts [3.227 (4) 3.401 (4) Å] are also observed in the crystal structure. The crystal studied was a non-morohedral twin with a 26.1 (6)% minor component.

## Related literature

For the structure of 2-bromo­nitro­benzene, see: Fronczek (2006 ▶). For the structure of 3-bromo­nitro­benzene, see: Charlton & Trotter (1963 ▶).
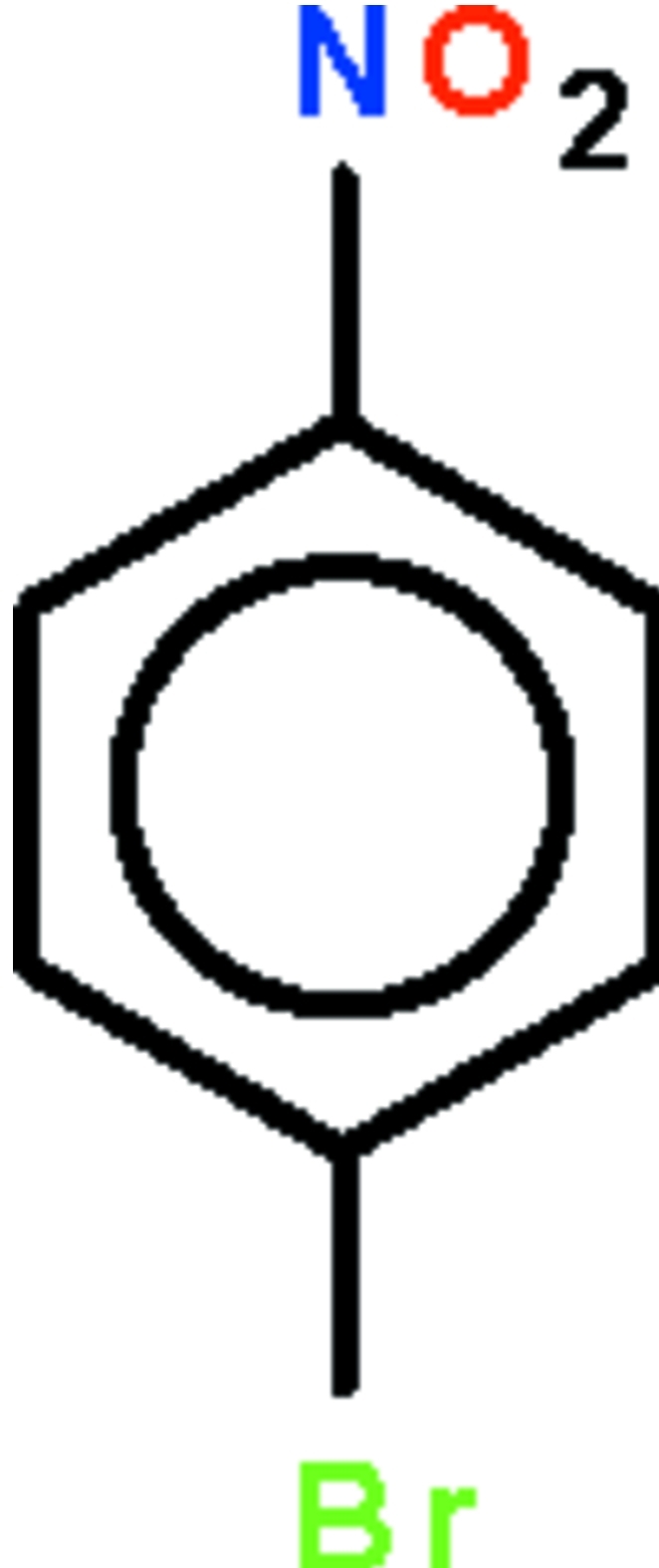


## Experimental

### 

#### Crystal data

C_6_H_4_BrNO_2_
*M* *_r_* = 202.01
Triclinic, 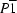

*a* = 6.3676 (6) Å
*b* = 7.3635 (7) Å
*c* = 7.6798 (7) Å
α = 65.554 (9)°
β = 87.705 (8)°
γ = 88.884 (8)°
*V* = 327.54 (5) Å^3^
*Z* = 2
Mo *K*α radiation
μ = 6.20 mm^−1^
*T* = 100 K
0.20 × 0.10 × 0.05 mm

#### Data collection

Agilent SuperNova Dual diffractometer with an Atlas detector
Absorption correction: multi-scan (*CrysAlis PRO*; Agilent, 2010 ▶) *T*_min_ = 0.414, *T*_max_ = 1.000
2142 measured reflections
1443 independent reflections
1365 reflections with *I* > 2σ(*I*)
*R*_int_ = 0.052

#### Refinement

*R*[*F* ^2^ > 2σ(*F* ^2^)] = 0.045
*wR*(*F* ^2^) = 0.119
*S* = 1.07
1443 reflections
92 parameters
H-atom parameters constrained
Δρ_max_ = 0.91 e Å^−3^
Δρ_min_ = −1.58 e Å^−3^

### 

Data collection: *CrysAlis PRO* (Agilent, 2010 ▶); cell refinement: *CrysAlis PRO*; data reduction: *CrysAlis PRO*; program(s) used to solve structure: *SHELXS97* (Sheldrick, 2008 ▶); program(s) used to refine structure: *SHELXL97* (Sheldrick, 2008 ▶); molecular graphics: *X-SEED* (Barbour, 2001 ▶); software used to prepare material for publication: *publCIF* (Westrip, 2010 ▶).

## Supplementary Material

Crystal structure: contains datablocks global, I. DOI: 10.1107/S1600536811003394/xu5150sup1.cif

Structure factors: contains datablocks I. DOI: 10.1107/S1600536811003394/xu5150Isup2.hkl

Additional supplementary materials:  crystallographic information; 3D view; checkCIF report

## Figures and Tables

**Table 1:** Hydrogen-bond geometry (Å, °)

*D*—H⋯*A*	*D*—H	H⋯*A*	*D*⋯*A*	*D*—H⋯*A*
C3—H3⋯O1^i^	0.95	2.52	3.359 (6)	147
C5—H5⋯O2^ii^	0.95	2.54	3.276 (6)	135

Symmetry codes: (i) 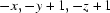; (ii) 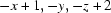.

**Fig. 1.**
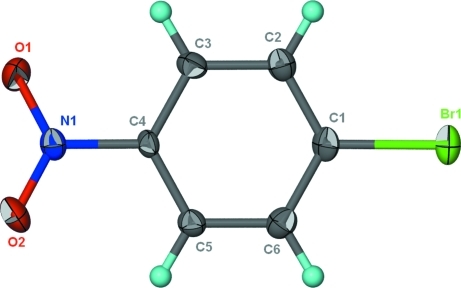
Thermal ellipsoid plot (Barbour, 2001) of C6H4BrNO2 at the 70% probability level; hydrogen atoms are drawn as spheres of arbitrary radius.

## supplementary crystallographic information

### Comment

4-Bromo-1-nitrobenzene (Scheme I) was synthesized as a precursor that will be used in the synthesis of 4,4'-bis(aminophenoxy)biphenyl (the compound is also commercially available: http://www.chemindustry.com/chemicals/815494.html). The molecule is flat (Fig. 1) as the nitro substituent is co-planar with the aromatic ring. π-π stacking occrs between parallel benzene rings of adjacent molecules, centroids distance between C1-ring and C1^i^-ring (symmetry code: (i) 1-x, -5, 1-z) is 3.643 (3) Å and that between C1-ring and C1^ii^-ring (symmetry code: (ii) 1-x, 1-y, 1-z) is 3.741 (3) Å. Intermolecular weak C—H···O hydrogen bonding (Table 1) and the short Br···O contacts [3.227 (4), 3.401 (4) Å] are observed in the crystal structure.

### Experimental

The nitrating mixture cosisted of 5 ml conc. HNO_3_ and 5 ml conc. H_2_SO_4_ kept at 273 K. Bromobenzene (2.6 ml) was added. The temperature was then raised to about 333 K for 3 h. The mixture was added to water (200 ml); the organic compound was extracted by using dichloromethane. The solvent was dried and then alllowed to evaporate to yield the product in 70% yield.

### Refinement

Carbon-bound H-atoms were placed in calculated positions [C—H 0.95 Å, *U*_iso_(H) 1.2*U*_eq_(C)] and were included in the refinement in the riding model approximation.

The crystal is a non-merohedral twin; the separation of the two domains was effected by *CrysAlis PRO* (Agilent, 2010).

### Crystal data

**Table:**

C_6_H_4_BrNO_2_	*Z* = 2
*M**_r_* = 202.01	*F*(000) = 196
Triclinic, *P*1	*D*_x_ = 2.048 Mg m^−^^3^
Hall symbol: -P 1	Mo *K*α radiation, λ = 0.71073 Å
*a* = 6.3676 (6) Å	Cell parameters from 1590 reflections
*b* = 7.3635 (7) Å	θ = 2.9–28.3°
*c* = 7.6798 (7) Å	µ = 6.20 mm^−^^1^
α = 65.554 (9)°	*T* = 100 K
β = 87.705 (8)°	Block, colorless
γ = 88.884 (8)°	0.20 × 0.10 × 0.05 mm
*V* = 327.54 (5) Å^3^	

### Data collection

**Table:**

Agilent SuperNova Dual diffractometer with an Atlas detector	1443 independent reflections
Radiation source: SuperNova (Mo) X-ray Source	1365 reflections with *I* > 2σ(*I*)
Mirror	*R*_int_ = 0.052
Detector resolution: 10.4041 pixels mm^-1^	θ_max_ = 27.5°, θ_min_ = 2.9°
ω scans	*h* = −8→8
Absorption correction: multi-scan (*CrysAlis PRO*; Agilent, 2010)	*k* = −9→9
*T*_min_ = 0.414, *T*_max_ = 1.000	*l* = −9→9
2142 measured reflections	

### Refinement

**Table:**

Refinement on *F*^2^	Primary atom site location: structure-invariant direct methods
Least-squares matrix: full	Secondary atom site location: difference Fourier map
*R*[*F*^2^ > 2σ(*F*^2^)] = 0.045	Hydrogen site location: inferred from neighbouring sites
*wR*(*F*^2^) = 0.119	H-atom parameters constrained
*S* = 1.07	*w* = 1/[σ^2^(*F*_o_^2^) + (0.0671*P*)^2^ + 0.4717*P*] where *P* = (*F*_o_^2^ + 2*F*_c_^2^)/3
1443 reflections	(Δ/σ)_max_ = 0.001
92 parameters	Δρ_max_ = 0.91 e Å^−^^3^
0 restraints	Δρ_min_ = −1.58 e Å^−^^3^

### Fractional atomic coordinates and isotropic or equivalent isotropic displacement parameters (Å^2^)

**Table:**

	*x*	*y*	*z*	*U*_iso_*/*U*_eq_	
Br1	0.83556 (7)	0.24763 (7)	0.14671 (6)	0.01978 (19)	
O1	0.0573 (5)	0.3167 (6)	0.7390 (5)	0.0238 (8)	
O2	0.3071 (6)	0.2117 (6)	0.9389 (5)	0.0245 (8)	
N1	0.2383 (6)	0.2623 (6)	0.7787 (5)	0.0151 (7)	
C6	0.7151 (7)	0.1751 (7)	0.5288 (7)	0.0173 (9)	
H6	0.8526	0.1212	0.5575	0.021*	
C5	0.5810 (7)	0.1811 (7)	0.6722 (6)	0.0146 (9)	
H5	0.6251	0.1326	0.8004	0.018*	
C2	0.4465 (7)	0.3256 (7)	0.2969 (7)	0.0173 (9)	
H2	0.4024	0.3750	0.1687	0.021*	
C3	0.3121 (7)	0.3291 (7)	0.4408 (6)	0.0159 (9)	
H3	0.1731	0.3792	0.4130	0.019*	
C1	0.6478 (7)	0.2483 (6)	0.3429 (6)	0.0157 (9)	
C4	0.3809 (7)	0.2594 (6)	0.6254 (6)	0.0125 (8)	

### Atomic displacement parameters (Å^2^)

**Table:**

	*U*^11^	*U*^22^	*U*^33^	*U*^12^	*U*^13^	*U*^23^
Br1	0.0204 (3)	0.0213 (3)	0.0206 (3)	−0.00244 (19)	0.00662 (18)	−0.0122 (2)
O1	0.0149 (16)	0.038 (2)	0.0232 (18)	0.0024 (15)	0.0001 (13)	−0.0174 (16)
O2	0.0299 (19)	0.032 (2)	0.0131 (16)	0.0028 (16)	−0.0003 (14)	−0.0107 (15)
N1	0.0173 (18)	0.0152 (18)	0.0156 (18)	−0.0013 (14)	0.0010 (14)	−0.0093 (15)
C6	0.015 (2)	0.017 (2)	0.021 (2)	−0.0008 (17)	0.0000 (17)	−0.0089 (19)
C5	0.016 (2)	0.015 (2)	0.014 (2)	0.0014 (16)	−0.0023 (16)	−0.0076 (17)
C2	0.020 (2)	0.018 (2)	0.016 (2)	−0.0004 (18)	0.0001 (17)	−0.0094 (18)
C3	0.018 (2)	0.016 (2)	0.015 (2)	0.0020 (17)	−0.0041 (17)	−0.0072 (17)
C1	0.019 (2)	0.014 (2)	0.019 (2)	−0.0005 (18)	0.0015 (18)	−0.0113 (19)
C4	0.0133 (19)	0.015 (2)	0.012 (2)	−0.0005 (16)	0.0005 (15)	−0.0090 (17)

### Geometric parameters (Å, °)

**Table:**

Br1—C1	1.887 (4)	C5—C4	1.387 (6)
O1—N1	1.220 (5)	C5—H5	0.9500
O2—N1	1.226 (5)	C2—C3	1.379 (6)
N1—C4	1.464 (5)	C2—C1	1.390 (6)
C6—C1	1.384 (6)	C2—H2	0.9500
C6—C5	1.381 (6)	C3—C4	1.380 (6)
C6—H6	0.9500	C3—H3	0.9500
			
O1—N1—O2	123.6 (4)	C1—C2—H2	120.6
O1—N1—C4	117.9 (4)	C4—C3—C2	119.6 (4)
O2—N1—C4	118.4 (4)	C4—C3—H3	120.2
C1—C6—C5	119.5 (4)	C2—C3—H3	120.2
C1—C6—H6	120.2	C6—C1—C2	121.5 (4)
C5—C6—H6	120.2	C6—C1—Br1	119.2 (3)
C4—C5—C6	118.7 (4)	C2—C1—Br1	119.3 (3)
C4—C5—H5	120.6	C3—C4—C5	121.8 (4)
C6—C5—H5	120.6	C3—C4—N1	119.7 (4)
C3—C2—C1	118.8 (4)	C5—C4—N1	118.4 (4)
C3—C2—H2	120.6		
			
C1—C6—C5—C4	0.5 (7)	C2—C3—C4—N1	−179.8 (4)
C1—C2—C3—C4	1.1 (7)	C6—C5—C4—C3	0.7 (7)
C5—C6—C1—C2	−0.9 (7)	C6—C5—C4—N1	179.0 (4)
C5—C6—C1—Br1	177.9 (3)	O1—N1—C4—C3	4.1 (6)
C3—C2—C1—C6	0.1 (7)	O2—N1—C4—C3	−175.3 (4)
C3—C2—C1—Br1	−178.7 (3)	O1—N1—C4—C5	−174.3 (4)
C2—C3—C4—C5	−1.5 (7)	O2—N1—C4—C5	6.3 (6)

### Hydrogen-bond geometry (Å, °)

**Table:**

*D*—H···*A*	*D*—H	H···*A*	*D*···*A*	*D*—H···*A*
C3—H3···O1^i^	0.95	2.52	3.359 (6)	147
C5—H5···O2^ii^	0.95	2.54	3.276 (6)	135

Symmetry codes: (i) −*x*, −*y*+1, −*z*+1; (ii) −*x*+1, −*y*, −*z*+2.

## References

[bb1] Agilent (2010). *CrysAlis PRO* Agilent Technologies, Yarnton, England.

[bb2] Barbour, L. J. (2001). *J. Supramol. Chem.* **1**, 189–191.

[bb3] Charlton, T. L. & Trotter, J. (1963). *Acta Cryst.* **16**, 313.

[bb4] Fronczek, F. R. (2006). Private communication (refcode 264855). CCDC, Cambridge, England.

[bb5] Sheldrick, G. M. (2008). *Acta Cryst.* A**64**, 112–122. 10.1107/S0108767307043930 18156677

[bb6] Westrip, S. P. (2010). *J. Appl. Cryst.* **43**, 920–925.

